# *In situ* Measuring Film-Depth-Dependent Light Absorption Spectra for Organic Photovoltaics

**DOI:** 10.3389/fchem.2020.00211

**Published:** 2020-04-07

**Authors:** Xiang Feng, Yuheng Wang, Tong Xiao, Zichao Shen, Yurong Ren, Guanghao Lu, Laju Bu

**Affiliations:** ^1^Frontier Institute of Science and Technology, and School of Science, Xi'an Jiaotong University, Xi'an, China; ^2^State Key Laboratory of Electrical Insulation and Power Equipment, Xi'an Jiaotong University, Xi'an, China

**Keywords:** organic photovoltaics, organic solar cells, bulk heterojunction, depth profiling, light absorption spectroscopy

## Abstract

Organic donor–acceptor bulk heterojunction are attracting wide interests for solar cell applications due to solution processability, mechanical flexibility, and low cost. The photovoltaic performance of such thin film is strongly dependent on vertical phase separation of each component. Although film-depth-dependent light absorption spectra measured by non-*in situ* methods have been used to investigate the film-depth profiling of organic semiconducting thin films, the *in situ* measurement is still not well-resolved. In this work, we propose an *in situ* measurement method in combination with a self-developed *in situ* instrument, which integrates a capacitive coupled plasma generator, a light source, and a spectrometer. This *in situ* method and instrument are easily accessible and easily equipped in laboratories of the organic electronics, which could be used to conveniently investigate the film-depth-dependent optical and electronic properties.

## Introduction

As one of the effective approaches to utilize solar energy converting energy, photovoltaics have received extensive attention. Achieving high-efficiency, large-area manufacture, and excellent environmental stability are the goals (Liu et al., 2015; Niu et al., 2018; Fan et al., 2019; Xu et al., 2019a,b; Zhu et al., 2019; Zhang et al., 2020). Especially, organic photovoltaic devices (OPVs) using solution-processible donor–acceptor bulk heterojunction (BHJ) as photon-harvesting active layer are attractive for next-generation flexible photovoltaic modules (Li et al., 2012, 2018; Lin et al., 2012; Heeger, 2014; Dong et al., 2019). With the development of organic optoelectronic materials, device structures, and fabrication processes, device performance has been fundamentally optimized, and power conversion efficiency (PCE) of single-junction OPV has exceeded 18% until now (Lin et al., 2015, 2019; Zhao et al., 2015, 2017; Liu et al., 2016, 2020; Meng et al., 2018; Cheng et al., 2019; Yan T. et al., 2019; Yuan et al., 2019). As compared with organic films deposited upon vacuum evaporation, during the BHJ films deposition from solution, the phase evolution of binary or ternary blends usually induces vertical phase separation, leading to film-depth-dependent distribution of both composition and morphology (Xu et al., 2009; Yan et al., 2017; Bi et al., 2018; Huang et al., 2018; Adil et al., 2019; Yan H. et al., 2019). To study the vertical distribution characteristics of film, many methods have been developed. However, the inherent disadvantages of these technologies limit further application, such as high cost, complicated operation, low preciseness, etc. For example, sputter combined with dynamic secondary ion mass spectroscopy is a universal method to study vertical component distribution by measuring element distribution in depth profile. However, this method is rather expensive. Additionally, high energy sputter treatment may damage the film, and primary ions also cause the surface state to change. Neutron reflectivity requires expensive equipment, namely, accelerator, which is not immediately accessible. The result accuracy of variable-angle spectroscopic ellipsometry is closely related to the fitting model, which requires multiple measurements to reduce errors. As for the three-dimensional imaging transmission electron microscope, the sample preparation process is complicated. Therefore, it is necessary to develop a simple and efficient method to study the film-depth-dependent characteristics.

BHJ solar cells are actually composed of anode/cathode, hole/electron transport layers, and photo-response active layer. Owing to light interferences among these layers, which have different refractive indices and extinction coefficients, the photon-harvesting profile is non-linear along vertical direction (Gusain et al., 2019). On the one hand, vertical phase separation between the donor and acceptor makes this photon-harvesting scenario more complicated, pointing to the importance of film-depth optical variations. After fission of exciton, the hole and electron need to transport along different pathways, i.e., donor and acceptor phases, toward anode and cathode, respectively. Therefore, the film-depth-dependent distribution of donor and acceptor plays an important role in device performance. On the other hand, the UV–visible (UV-vis) light absorption is caused by the transition of valence electrons to the conductance band, while thus formed hole–electron pair has a so-called binding energy. Consequently, for organic semiconductors, the light absorption spectra could roughly reflect the electronic properties of organic materials. By this way, we can indirectly investigate the charge behavior in the active layer (Bredas, 2014a,b). Therefore, UV-vis light absorption spectroscopy is a commonly used characterization method for optical and electronic properties of BHJ photovoltaic layers. Although depth profiling upon film-depth-dependent light absorption spectra (FLAS) has been previously developed by us and successfully applied in polymer solar cells and organic thin-film transistors (Bu et al., 2016a,b; Wang J. et al., 2018; Liang et al., 2019), the non-*in situ* measurement is time consuming, less precise, and difficult to be integrated with other measurements.

In this work, we propose an *in situ* measurement method in combination with an *in situ* instrument that integrates a capacitive coupled plasma generator, a light source, and a spectrometer to *in situ* measure FLAS. The *in situ* FLAS of organic BHJ active layer could be measured more immediately within a few minutes, with improved film depth resolution on the scale of nanometer, as compared with non-*in situ* measurements. Subsequently, these *in situ* FLAS are used to investigate vertical distribution of composition and aggregation, the photon harvesting profile along film-depth direction, and film-depth-dependent charge transport behavior.

## Materials and Methods

### Materials

The indium tin oxide (ITO) glass substrates with sheet resistance of ≤ 15 Ω sq^−1^ were purchased from South China Science& Technology Co., Ltd. The poly{(2,6-(4,8-bis(5-(2-ethylhexyl)thiophen-2-yl)-benzo[1,2-b:4,5-b′]dithiophene))-alt-(5,5-(1′,3′-di-2-thienyl-5′,7′-bis(2-ethylhexyl)benzo[1′,2′-c:4′,5′-c′]dithiophene-4,8-dione))} (PBDB-T) was purchased from Organtec Ltd. The 3,9-bis{2-methylene-[3-(1,1-dicyanomethylene)-indanone]}-5,5,11,11-tetrakis(4-hexylphenyl)-dithieno[2,3-d:2′,3′-d′]-s-indaceno[1,2-b:5,6-b′]dithiophene (ITIC) was purchased from Solarmer Materials Inc. The zinc acetate dihydrate [Zn(CH_3_COO)_2_·2H_2_O], ethanolamine (NH_2_CH_2_CH_2_OH), 2-methoxyethanol (CH_3_OCH_2_CH_2_OH), and polystyrene (PS) were purchased from Sigma-Aldrich Inc. The molecular weight of PS was 2,000 K with polymer dispersity index of 1.3. The molybdenum trioxide (MoO_3_) was purchased from Adamas Reagent Co., Ltd. The poly(3,4-ethylenedioxythiophene):poly(styrenesulfonate) (PEDOT:PSS) was purchased from Xi'an Polymer Light Technology Corp.

### Fabrication and Measurement of BHJ Devices

The device structure was ITO/ZnO/active layer/MoO_3_/Al. The ITO substrates were ultrasonically cleaned by detergent, deionized water, acetone, and isopropanol for 15 min, respectively. After being dried with nitrogen, the substrates were treated with UV ozone for 30 min. The zinc oxide (ZnO) precursor solution was obtained by dissolving 1 g Zn(CH_3_COO)_2_·2H_2_O and 0.28 g NH_2_CH_2_CH_2_OH into 10 ml CH_3_OCH_2_CH_2_OH, and then spin coated at 3,000 rpm. The ITO substrates were baked at 150°C for 15 min to form the ZnO layer (30 nm) and then transferred to nitrogen glove box. The PBDB-T and ITIC (*w*/*w* = 1:1) were dissolved in chlorobenzene at the concentration of 20 mg/ml. The active layer solution was spin coated on the ZnO layer at 2,000 rpm. In the end, hole transport layer MoO_3_ (10 nm) and electrode Al (100 nm) were deposited by vacuum evaporation, respectively. The device effective area was 0.04 cm^2^. The AM 1.5G solar simulator (light intensity, 100 mW/cm^2^) was used as the light source for *J*–*V* characteristic measurement.

### Fabrication of Bilayer Configuration Films

The bilayer configurations of PBDB-T/ITIC and ITIC/PBDB-T were fabricated through layer-by-layer transferring method. The ITO substrates were ultrasonically cleaned by detergent, deionized water, acetone, and isopropanol for 15 min, respectively. The PBDB-T and ITIC were dissolved in chlorobenzene at the concentration of 20 mg/ml, respectively. To facilitate the transfer of ITIC film, the blend solution of ITIC and PS (*w*/*w* = 2:1) was obtained in chlorobenzene at the concentration of 20 mg/ml. As for PBDB-T/ITIC film, PEDOT:PSS was spin coated on the substrate at 5,000 rpm; then, PBDB-T solution was spin coated at 2,000 rpm. The substrate was put into deionized water slowly, and PBDB-T film could float due to the water solubility of PEDOT:PSS. The floated PBDB-T film was transferred onto the substrate with spin-coated ITIC film at 2,000 rpm. The fabrication of ITIC/PBDB-T film was almost similar, and the difference was that blend solution of ITIC and PS was spin coated on the PEDOT:PSS layer.

### Film-Depth-Dependent Light Absorption Spectra Measurement

The FLAS measurement uses the instrument setup by our laboratory, which will be described in detail in the following parts. The sample was put into the capacitive coupled plasma generator (PT-5S, Shenzhen Sanwa Boda Mechanical & Electrical Technology Co., Ltd.), and the vacuum pump (2XZ-4, Shenzhen Sanwa Boda Mechanical & Electrical Technology Co., Ltd.) evacuated to ~3 Pa. Then, oxygen entered into vacuum discharge chamber, and plasma was generated by capacitive coupling discharge etching the film. The spectrometer (PG2000-Pro, Ideaoptics) collected the *in situ* spectrum information of film after each etching. Each etching time is almost identical (~20 s).

## Results and Discussion

### *In situ* Instrument and *in situ* Spectra Measurement

**Figure 1:**
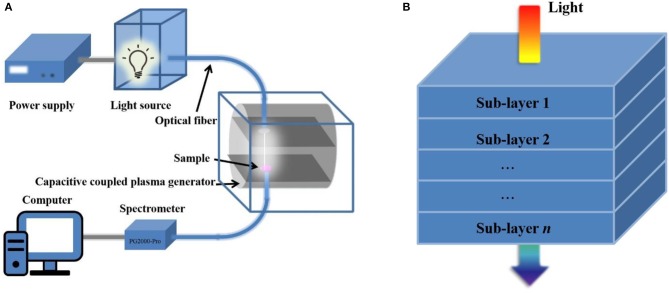
Schemes of instrument and analytical principle for *in situ* measuring FLAS. **(A)** Components of the instrument. **(B)** Schematic diagram showing the layer is composed of *n* sublay.

As shown in Figure 1A, the *in situ* instrument is mainly composed of a power supply, a light source, a capacitive coupled plasma generator, a spectrometer, and a computer. The power supply can provide the stable constant current to ensure the continuous work of light source. The light source is a xenon lamp (150 W), and its spectrum is from ultraviolet to near infrared. To reduce the impact of output power density loss and ensure the constant output, light intensity is adjustable and output characteristics of power supply are required. The stable current can be adjusted in the range of 7–9 A; meanwhile, the current instability is limited to 0.05%. The spectrometer provides balanced sensitivity and high resolution within the spectrum of 200–1,100 nm. The Morpho, as spectrometer analysis software, displays the spectra in real time.

The integration of plasma etching and *in situ* spectra measurement is realized by introducing the optical path into the capacitive coupled plasma generator. The plasma generator selects a sealed low dielectric vacuum discharge chamber made of aluminum alloy to approximate the total reflection integrating sphere. The optical fiber is tightly connected to the upper and lower sides of vacuum discharge chamber by fastener to ensure the vacuum degree and avoid the movement that may deteriorate the focus of optical path. When the *in situ* instrument works, the divergent light from xenon lamp becomes parallel through a plano-convex lens and enters the optical fiber after gathering. The light is irradiated to the sample through optical fiber above the vacuum chamber and then enters the spectrometer through optical fiber below the vacuum chamber. After light is processed by a spectrometer and spectrum analysis software, the absorption spectrum is obtained. The sample is always fixed at the same position during etching and measurement and continuously thinned by the oxygen plasma. Considering the effect of different etching rate, this *in situ* measurement method is suitable for organic film with small phase separation scale and surface roughness. To achieve outstanding device performance, most organic optoelectronic films are eligible. Additionally, due to the low etching power and short treatment time, temperature in the vacuum chamber does not rise too much, and it is just slightly higher than room temperature during the entire etching process, which cannot cause film nanostructure to evolve.

Previous research has proven that the plasma can selectively etch the organic film without damaging the materials below the surface at low pressure (<30 Pa) (Bu et al., 2016a). Hence, we can etch the film with low-pressure plasma and measure the *in situ* absorption spectra to investigate the film-depth-dependent characteristics. The theoretical basis for obtaining the FLAS is based on Lambert–Beer law. As follows:

(1) $$\begin{array}{l} A=-\log\frac{I}{I_{0}-I_{R}} \end{array}$$

(2) $$\begin{array}{l} I=\left(I_{0}-I_{R}\right)10^{-A} \end{array}$$

where *A* is the absorbance; *I*_0_, *I*, and *I*_*R*_ are the incident, transmitted, and reflected light intensity, respectively. It is obvious that the proportion of light absorption by equal thickness layer is identical regardless of incident light intensity. In this case, if the active layer is assumed to be divided into *n* sublayers, and the absorbance of each sublayer is *A*_*i*_ (*i* = 1, 2, 3,…, *n*) (Figure 1B), then the transmitted light intensity is

(3) $$\begin{array}{l} I=\left(I_{0}-I_{R}\right)\overset{n}{\underset{i=1}{\prod }}10^{{-A}_{i}}=\left(I_{0}-I_{R}\right)10^{-\sum_{i}^{n}A_{i}} \end{array}$$

(4) $$\begin{array}{l} A_{\text{active layer}}=A_{1}+A_{2}+\ldots +A_{n} \end{array}$$

It can be seen that the absorption spectrum of film can be considered as a linear superposition of the sublayers. Thus, the FLAS can be obtained by subtracting the absorption spectrum of etching film layer by layer.

**Figure 2:**
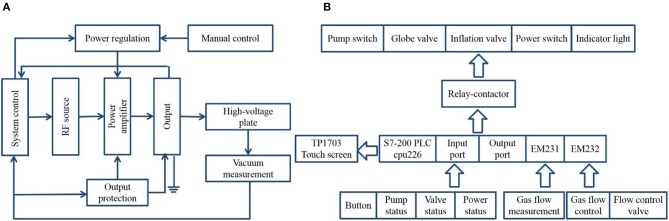
Control principles of capacitive coupled plasma generator. **(A)** System working flow chart. **(B)** Programmable logic controller (PLC) control system framework.

As the most important part of entire instrument, we focus on the capacitive coupled plasma generator. It mainly includes three parts: radio frequency (RF) power supply part, system control part, and vacuum and gas circuit part. Owing to the small RF power, the parts of RF power supply and system control can be assembled to save room and simplified the operation. Figure 2A shows the system working flow chart of capacitive coupled plasma generator. With the set vacuum degree, the circuit system starts the RF source to generate a 40-kHz RF pulse (offset is <0.2 kHz), and then, RF pulse is amplified to the output stage. To ensure that it has enough energy to excite the high-voltage plate and generate plasma, the role of output stage is 3-fold: isolation, boost, and matching.

The RF power is adjusted by changing the width of RF pulse, which can be set in the system or manual control, and it is generally 300 W during the measurement. The ideal RF pulse output does not consider the loss of components and magnetic materials, but the actual RF pulse waveform is distorted. When the distortion is severe, the effective power output can be greatly reduced. In addition, excessive harmonic pulse can also reduce the RF efficiency, which can not only cause abnormal plasma generation but also burn the equipment. Thus, the technical demands for RF power are required. Figure 2B shows the frame of control system. According to the control requirements of capacitive coupled plasma generator, the S7-200 series programmable logic controller (PLC) with 24 digital input terminals, 16 digital output terminals, and 2 RS485 communication/programming ports is selected, which has compact structure, strong scalability, and rich instruction. The analog input (EM231) and analog output (EM232) modules are expanded to control and detect the gas flow. The TP170B touch screen can realize the etching parameter setting, process monitoring, and display functions of entire system. The gas inlet and outlet ports are set at the back of capacitive coupled plasma generator. The two gas inlet ports are connected to gas source, and the gas outlet port is connected to vacuum pump. The type and mixing ratio of gas can be selected through the touch screen. To control pressure of vacuum discharge chamber, gas flow can be adjusted by the pressure reducing valve of gas cylinder and flow meter.

### Glow Discharge Emission Spectra at Different Oxygen Plasma Pressures

**Figure 3:**
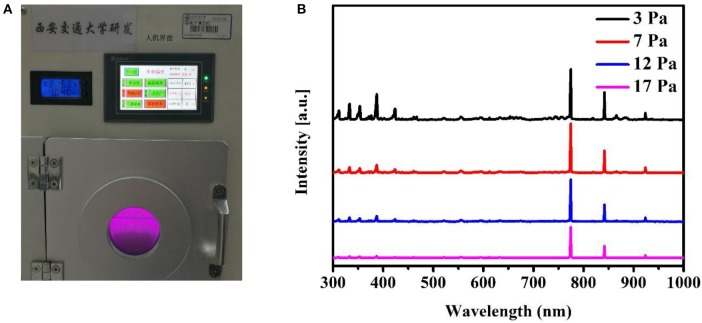
Glow discharge generating plasma. **(A)** A photo of the *in situ* film-depth-dependent light absorption spectra (FLAS) instrument during the oxygen plasma generation. The purple area in the photo shows oxygen plasma glow discharge. **(B)** Emission spectra of oxygen plasma during glow discharge at different oxygen plasma pressures. The spectra are shifted along the vertical direction for clarity.

The discharge mode of plasma generator is RF glow discharge with capacitive coupling. Compared with traditional direct current glow discharge, RF glow discharge can process insulated samples (Conrads and Schmidt, 2000). At low pressure, gas is ionized in an alternating electric field between the high-voltage plates. The charged ions are accelerated to continuously collide with molecules, and the plasma is generated. At the same time, the unstable excited-state atoms can return to ground states and emit photons in a short time, releasing energy in the form of light to form the glow. According to different gas categories, colorful visible light from blue to deep purple can be emitted, and the material processing temperature is close to room temperature. The selected gas source is oxygen, and the color of oxygen plasma glow discharge is purple (Figure 3A). Oxygen plasma contains a variety of reactive components, including O^2+^, O^2−^, O_3_, O, O^+^, O^−^, ionized ozone, etc. These highly active particles interact with the treated surface to get the etched surface modification (Chan et al., 1996).

As we have known, gas pressure can affect the intensity of glow discharge and further affect the etching rate. We study the oxygen plasma glow discharge spectra at different pressures. Owing to the complex plasma composition, emission spectra include the emission of molecules and atoms. The sharp emission peaks are mainly at 842 nm [OI (3p3P−3s3S)] and 774 nm [OI (3p5P−3s5S)] (Vandsburger et al., 2013). The emission band at 300–400 nm mainly comes from the emission of O [OII (3p−3s) at 423 nm and OIII (3d−3p) at 332 nm], OH, and N2 as a result of the presence of air. As shown in Figure 3B, the lower the pressure, the stronger the intensity of glow discharge spectrum, which means the faster the etching rate. Generally, the etching pressure is maintained at ~3 Pa to ensure that the material below the surface is not damaged. At the same time, the etching rate is maintained at a fast level.

### Device Performance and Film-Depth-Dependent Light Absorption Spectra

**Figure 4:**
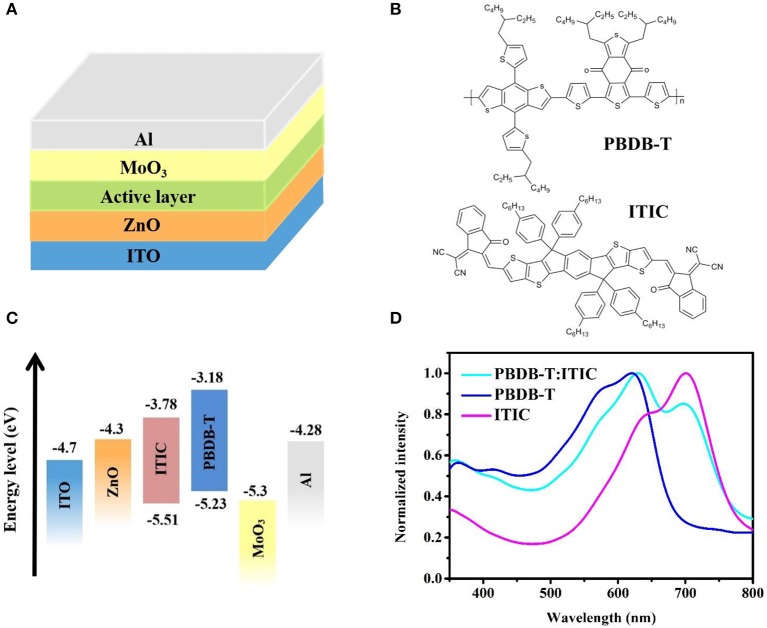
**(A)** Inverted device structure of organic photovoltaic devices (OPVs) studied in this work. **(B)** Molecular structures of poly{(2,6-(4,8-bis(5-(2-ethylhexyl)thiophen-2-yl)-benzo[1,2-b:4,5-b′]dithiophene))-alt-(5,5-(1′,3′-di-2-thienyl-5′,7′-bis(2-ethylhexyl)benzo[1′,2′-c:4′,5′-c′]dithiophene-4,8-dione))} (PBDB-T) and 3,9-bis{2-methylene-[3-(1,1-dicyanomethylene)-indanone]}-5,5,11,11-tetrakis(4-hexylphenyl)-dithieno[2,3-d:2′,3′-d′]-s-indaceno[1,2-b:5,6-b′]dithiophene (ITIC). **(C)** Energy level diagram of individual layers in OPVs. **(D)** Normalized absorption spectra of neat PBDB-T, neat ITIC, and PBDB-T:ITIC blend film.

The inverted device structure studied in this work is ITO/ZnO/Active layer/MoO_3_/Al (Figure 4A), with PBDB-T as donor and ITIC as acceptor. Compared with standard structure, the air stability of inverted structure is better. Figure 4B shows the molecular structures of PBDB-T and ITIC. Figure 4C shows the energy levels of individual layers in OPVs from the literature (Li et al., 2019).

Figure 4D shows the absorption spectra of neat PBDB-T film, neat ITIC film, and blend film. The absorption spectrum of PDBD-T is mainly at 500–700 nm, and the absorption spectrum of ITIC ranges from 550 to 800 nm. The absorption spectrum of blend film is from 500 to 800 nm.

**Figure 5:**
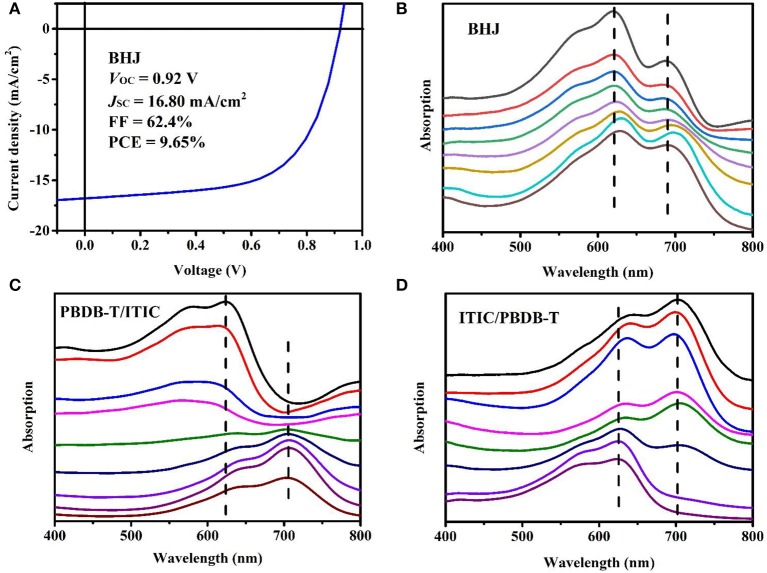
Device performance and film-depth-dependent light absorption spectra (FLAS). **(A)** *J–V* characteristic curve of bulk heterojunction (BHJ) device under illumination of solar simulator (AM 1.5G, 100 mW/cm^2^). **(B–D)** FLAS of BHJ and bilayer. **(B)** Poly{(2,6-(4,8-bis(5-(2-ethylhexyl)thiophen-2-yl)-benzo[1,2-b:4,5-b′]dithiophene))-alt-(5,5-(1′,3′-di-2-thienyl-5′,7′-bis(2-ethylhexyl)benzo[1′,2′-c:4′,5′-c′]dithiophene-4,8-dione))}:3,9-bis{2-methylene-[3-(1,1-dicyanomethylene)-indanone]}-5,5,11,11-tetrakis(4-hexylphenyl)-dithieno[2,3-d:2′,3′-d′]-s-indaceno[1,2-b:5,6-b′]dithiophene (PBDB-T:ITIC) (1:1) BHJ. The dashed lines show that the absorption peak varies with film depth. **(C)** PBDB-T/ITIC bilayer configuration with PBDB-T layer at the top and ITIC layer at the bottom, as prepared by layer-by-layer transferring method. **(D)** ITIC/PBDB-T bilayer configuration with ITIC layer at the top and PBDB-T layer at the bottom. ITIC was blended with some polystyrene to warrant the film transferring. The upper sublayer spectrum corresponds to the top of film, and the lower sublayer spectrum corresponds to the bottom of film. The spectra are shifted along the vertical direction for clarity. The sublayer thickness corresponding to each spectrum is ~12–13 nm.

As a classic system, lots of work were devoted to performance optimization and mechanism research of PBDB-T/ITIC devices (Zhao W. et al., 2016; Zhao W. C. et al., 2016; Pan et al., 2017; An et al., 2018; Bi et al., 2018; Liang et al., 2018). Here, we mainly study the film-depth-dependent optical and electrical properties by the *in situ* instrument. Without additive and annealing treatment, the as-cast devices show good device performance. From the *J–V* characteristic curve of BHJ device (Figure 5A), the PCE of 9.65% was achieved, with the open circuit voltage (*V*_oc_) of 0.92 V, short circuit current density (*J*_sc_) of 16.80 mA/cm^2^, and fill factor (FF) of 62.4%. The UV-vis absorption spectra are sensitive to chain conformation and aggregation structure of conjugated organic material. As for PBDB-T /ITIC, the π–π stacking between molecular chains leads to the delocalization of π electrons, which reduces the optical gap. In other words, the red shift of absorption peak means more ordered molecular aggregation. From the FLAS of BHJ (Figure 5B), the first sublayer shows red shift compared to the second sublayer, indicating the better molecular packing at the top surface of the film. The difference in aggregate structure along the vertical direction is strongly correlated with the solvent evaporation process.

To demonstrate the validity of measurement, bilayer configuration films were prepared for comparison. We prepared two kinds of structures: one is PBDB-T/ITIC, and the other is ITIC/PBDB-T from top to bottom. The thickness of single-layer films were ~100 nm, so the thickness of bilayer configuration films were ~200 nm. As for ITIC/PBDB-T structure, little PS was added to the ITIC solution to facilitate the transfer of small molecule ITIC film. In addition, the absorption peak of PS is at 190 nm, which do not affect the absorption spectrum of ITIC. We did not measure the performance of bilayer configuration devices because the films made by this method were rough, resulting in poor device performance. As shown in Figures 5C, D, the FLAS of bilayer configuration films show significant stratification. As for PBDB-T/ITIC film, the FLAS on the top mainly show the peak of PBDB-T, while the FLAS on the bottom mainly show the peak of ITIC. The ITIC/PBDB-T film is just the opposite. Moreover, due to structural difference, donor and acceptor materials are etched by oxygen plasma at different rates, but the difference is not so great.

### Film-Depth-Dependent Optical and Electronic Properties

**Figure 6:**
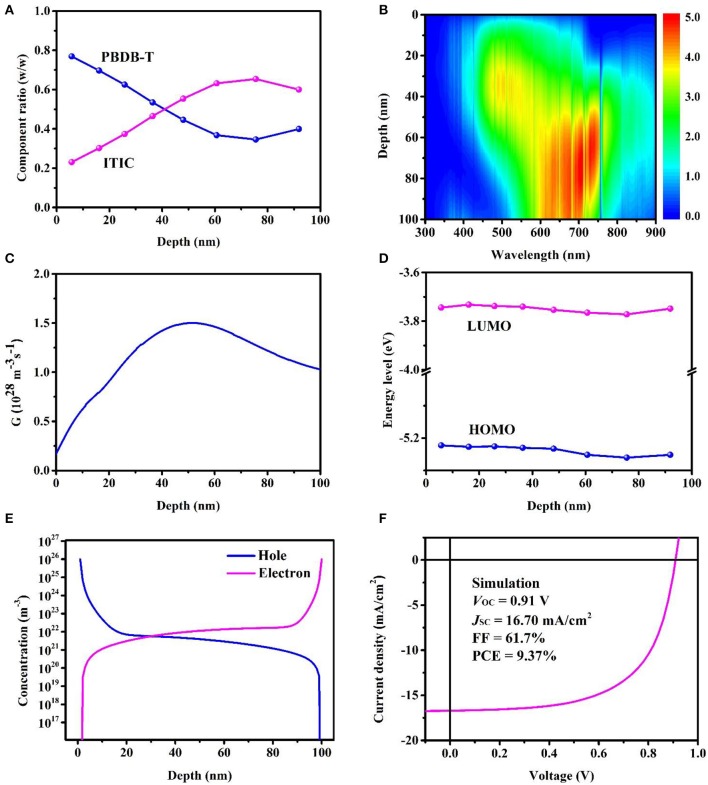
Film-depth-dependent optical and electronic properties of bulk heterojunction (BHJ) devices; 0 and 100 nm refer to active layer/MoO_3_ and ZnO/active layer interfaces, respectively. **(A)** Composition distribution profiles of donor and acceptor, as extracted from film-depth-dependent light absorption spectra (FLAS). **(B)** Exciton generation contour at wavelength and film depth direction (unit, 10^25^ m^−3^ s^−1^ nm^−1^). The noise-like vertical lines are caused by the fluctuation of real solar spectrum (AM 1.5G). **(C)** Exciton generation profile at film depth direction calculated from **(B)**. **(D)** Film-depth-dependent energy levels {highest occupied molecular orbital (HOMO) of Poly{(2,6-(4,8-bis(5-(2-ethylhexyl)thiophen-2-yl)-benzo[1,2-b:4,5-b′]dithiophene))-alt-(5,5-(1′,3′-di-2-thienyl-5′,7′-bis(2-ethylhexyl)benzo[1′,2′-c:4′,5′-c′]dithiophene-4,8-dione))} (PBDB-T) and lowest unoccupied molecular orbital (LUMO) of 3,9-bis{2-methylene-[3-(1,1-dicyanomethylene)-indanone]}-5,5,11,11-tetrakis(4-hexylphenyl)-dithieno[2,3-d:2′,3′-d′]-s-indaceno[1,2-b:5,6-b′]dithiophene (ITIC)}, as approximately obtained from FLAS. **(E)** Simulated film-depth-dependent carrier concentration distribution. **(F)** Simulated *J-V* characteristic curve, using film-depth-dependent optical **(B)** and electronic **(D)** properties, both of which are extracted from FLAS.

Numerical simulation is an effective method to research the physical mechanism and predict the theoretical limit efficiency of OPVs (Zhao et al., 2011; Kirchartz and Nelson, 2014). Utilizing the FLAS, we can investigate the film-depth-dependent optical and electronic properties of organic film by appropriate models and further apply to device performance optimization. Comparing the pure composition absorption spectra and FLAS, we fit the thickness and composition distribution of each sublayer along film-depth direction by least squares method. As shown in Figure 6A, the active layer forms vertical phase separation. The donor aggregates on the top and the acceptor aggregates on the bottom, which is beneficial to the inverted device. After exciton dissociation, the holes and electrons can be quickly collected by anode and cathode, respectively.

The transfer matrix method, considering the interference of light between multilayer film, has been widely used in the optical simulation of OPVs to investigate photoelectric field and exciton generation rate distribution (Pettersson et al., 1999; Xia et al., 2019). In this model, complex refractive index, *N* = *n* + *ik*, is the main optical constant, where *n* is the refractive index and *k* is the extinction coefficient. The refractive index *n* has little effect on the light absorption, and the refractive index of polymer is almost 2, so it can be directly taken as 2 to simplify calculation. The extinction coefficient is obtained from the FLAS according to

(5) $$\begin{array}{l} A=-\mathrm{lg}\left(e^{-\frac{4\pi kd}{\lambda }}\right) \end{array}$$

where *d* is the calculated sublayer thickness, and λ is the wavelength. Considering the vertical distribution of composition, we divide the active layer into multiple sublayers. Each sublayer takes a different extinction coefficient, and its interior is considered to be uniform (Wang Y. et al., 2018). Then, the exciton generation contour is obtained (Figure 6B), and exciton generation profile is calculated by integrating the wavelengths from the exciton generation contour (Figure 6C). The positions of exciton generation are mainly located in the middle of active layer, and sunlight with the range from 500 to 800 nm contributes the most, which is consistent with the absorption spectra of donor and acceptor. It is beneficial that excitons are generated and separated in the middle of the active layer for OPVs. In this way, the distance of charge transport to electrode is short, which means a smaller probability of recombination, resulting in higher PCE.

Owing to the complex molecular conformation and chain orientation of conjugated polymer, the energy band structure is significantly different from that of inorganic material (Gregg and Hanna, 2003). The energy levels for charge transport are closely associated with highest occupied molecular orbital (HOMO) and lowest unoccupied molecular orbital (LUMO) levels. Because of vertical phase separation, the molecular aggregation along the film-depth direction is different, further causing fluctuation of energy band (Blakesley and Neher, 2011). Figure 6D shows film-depth-dependent energy levels (HOMO of PBDB-T and LUMO of ITIC) extracted from FLAS. As follows:

(6) $$\begin{array}{l} E_{\text{LUMO}}=E_{A-\text{LUMO}}+\left(\frac{1,240}{\lambda_{\text{A}-\text{FLAS}}}-\frac{1,240}{\lambda_{\text{A}}}\right) \end{array}$$

(7) $$\begin{array}{l} E_{\text{HOMO}}=E_{\text{D}-\text{HOMO}}+\left(\frac{1,240}{\lambda_{\text{D}-\text{FLAS}}}-\frac{1,240}{\lambda_{\text{D}}}\right) \end{array}$$

where *E*_A−LUMO_ and *E*_D−HOMO_ are the LUMO of acceptor and HOMO of donor, respectively. λ_A_ and λ_D_ are the wavelengths corresponding to absorption peaks of acceptor and donor neat film, respectively. λ_A−FLAS_ and λ_D−FLAS_ are the wavelengths corresponding to absorption peaks of acceptor and donor FLAS, respectively. As we all know, the fluctuation of energy band is easy to form traps and leads to the recombination of carriers, which is detrimental to the device performance. Furthermore, based on drift-diffusion model, the effect of band fluctuation is introduced into potential term (Scheunemann et al., 2019; Wang et al., 2019; Xiao et al., 2020). The carrier concentration distribution is calculated (Figure 6E). Carriers gather in the middle of the active layer and deplete within a few nanometers at both interfaces. We also simulate the *J–V* curve of BHJ device, which is in good agreement with the experimental result (Figure 6F).

## Conclusions

In this work, we propose an *in situ* measurement method in combination with an *in situ* instrument that integrates a capacitive coupled plasma generator, a light source, and a spectrometer. This *in situ* method and instrument are easily accessible and easily equipped in laboratories. Subsequently, the *in situ* FLAS are used to investigate vertical distribution of composition and aggregation, photon harvesting contour along film-depth direction, and film-depth-dependent charge transport behavior. The *in situ* measurement and simulation contribute to the optimization of photovoltaic devices. This work provides a general method for *in situ* film-depth profiling, which could be used to conveniently investigate the film-depth-dependent optical and electronic properties.

## Data Availability Statement

All datasets generated for this study are included in the article/supplementary material.

## Author Contributions

XF performed the glow discharge spectra and FLAS measurement, simulated the optical and electronic properties of OPVs, and wrote the manuscript. YW and TX conducted the instrument construction and numerical model building. ZS fabricated the BHJ devices. YR revised the manuscript. LB and GL supervised the project and contributed to the writing of manuscript.

### Conflict of Interest

The authors declare that the research was conducted in the absence of any commercial or financial relationships that could be construed as a potential conflict of interest.
